# *De Novo* Glycine Synthesis Is Reduced in Adults With Morbid Obesity and Increases Following Bariatric Surgery

**DOI:** 10.3389/fendo.2022.900343

**Published:** 2022-06-09

**Authors:** Hong Chang Tan, Jean W. Hsu, E Shyong Tai, Shaji Chacko, Vieon Wu, Chun Fan Lee, Jean-Paul Kovalik, Farook Jahoor

**Affiliations:** ^1^ Department of Endocrinology, Singapore General Hospital, Singapore, Singapore; ^2^ Children’s Nutrition Research Center, Agricultural Research Service, U.S. Department of Agriculture, and Department of Pediatrics, Baylor College of Medicine, Houston, TX, United States; ^3^ Department of Medicine, Yong Loo Lin School of Medicine, National University Health System, Singapore, Singapore; ^4^ Centre of Quantitative Medicine, Duke-NUS Medical School, Singapore, Singapore; ^5^ Cardiovascular & Metabolic Disorders Program, Duke-NUS Medical School, Singapore, Singapore

**Keywords:** morbid obesity, bariatric surgery, *de novo* glycine synthesis, insulin resistancE, stable-isotope tracers, metabolic flux, substrate kinetics, glycine

## Abstract

**Background:**

Glycine is a dietary non-essential amino acid that is low in obesity and increases following bariatric surgery. However, the exact mechanism responsible remains unclear and it is unknown whether hypoglycinemia is a cause or consequence of insulin resistance.

**Objective:**

Using multiple isotopically labeled tracers, we aimed to determine the underlying kinetic changes responsible for hypoglycinemia in obesity by: 1) Comparing glycine kinetics between participants with morbid obesity (BMI ≥ 32.5 kg/m^2^) to those with healthy weight (BMI < 25 kg/m^2^), and 2) Comparing glycine kinetic changes in participants with morbid obesity after bariatric surgery.

**Methods:**

[1,2-^13^C_2_] glycine, [2,3,3-^2^H_3_] serine, and [^2^H_5_] phenylalanine were infused to compare the glycine kinetic parameters between 21 participants with morbid obesity and 21 controls with healthy weight. Participants with morbid obesity then underwent bariatric surgery and 17 were re-studied 6 months later. Data were analyzed by non-parametric methods and presented as median (interquartile range).

**Results:**

Compared to controls, participants with morbid obesity had significantly lower plasma glycine concentrations at 163 (153-171) vs. 201 (172-227) µmol/L and significantly reduced *de novo* glycine synthesis rate at 86.2 (64.5-111) vs.124 (103-159) µmol·kg LBM^-1^·h^1^, p < 0.001. Following surgery, body weight and insulin resistance decreased and this was accompanied by significant increases in plasma glycine concentration to 210 (191-243) µmol/L as well as the *de novo* glycine synthesis rate to 127 (98.3-133) µmol·kg LBM^-1^·h^-1^, p < 0.001 vs. baseline.

**Conclusion:**

Hypoglycinemia in participants with morbid obesity was associated with impaired *de novo* glycine synthesis. The increase in plasma glycine concentration and *de novo* glycine synthesis plus the marked improvement in insulin resistance after bariatric surgery suggest that hypoglycinemia may be secondary to impaired glycine synthesis because of obesity-induced insulin resistance.

**Clinical Trial Registration:**

[https://tinyurl.com/6wfj7yss], identifier [NCT04660513].

## Introduction

Obesity is a risk factor for insulin resistance and is associated with disturbances in the metabolism of not only glucose and lipids, but also of certain amino acids. Branched-chain amino acids (BCAAs), phenylalanine, and tyrosine are among the commonly reported amino acids with elevated plasma concentrations in individuals with obesity (1). Glycine, by contrast, has a lower plasma concentration in patients with obesity compared to those with a healthy weight (2) (3, 4). Higher plasma concentration of BCAAs in obesity has been attributed to either accelerated flux from protein breakdown (5, 6) or dysregulated BCAA clearance (7–9). However, the reasons why plasma glycine concentration is decreased in obesity constitute a metabolic enigma.

Compared to BCAAs, glycine is a nutritionally non-essential amino acid. This means that the human body can obtain its glycine requirement from dietary intake and through endogenous *de novo* synthesis (10). Yet, blood glycine concentration is lower in obesity, which is a state of overnutrition. At the same time, glycine is required in large quantities by the human body for the biosynthesis of physiologically important biomolecules, maintenance of oxidative and detoxification defenses, and growth and development (11, 12). Since obesity is a state of heightened metabolic demand, plasma glycine concentration can be low due to an increase in glycine catabolism or the diversion of glycine to utilization pathways (3, 4). However, the underlying kinetic changes in the metabolic pathways responsible for obesity-associated hypoglycinemia have not been well studied (3, 4).

Insulin resistance is the primary driver in the pathogenesis of type 2 diabetes, and weight reduction remains the primary method to lower insulin resistance in individuals with obesity. However, conventional lifestyle interventions often fail in the real world (13), and weight-loss medications can result in undesirable side effects (14). Bariatric surgery is the most effective treatment for individuals with morbid obesity, and patients also benefit from significant improvements in insulin resistance, diabetes control, and cardiovascular health (15). That said, not all patients are willing or suitable to undergo surgery. Therefore, a safe, effective, and well tolerated treatment against insulin resistance is urgently needed. Plasma glycine concentration correlates inversely with insulin resistance, and it has been speculated that the lower plasma glycine concentration in individuals with obesity worsens insulin resistance. Conversely, correcting hypoglycinemia may improve insulin resistance (3, 16). Hence, glycine-based treatment could be explored as a novel alternative or adjunctive therapy against insulin resistance.

To develop effective glycine-based treatments, there is a need to understand why glycine metabolism in dysregulated in obesity. If glycine “deficiency” is secondary to the body’s inability to synthesize glycine, then hypoglycinemia could be corrected through simple measures such as dietary supplementation. Conversely, if glycine deficiency is due to accelerated glycine utilization by catabolic pathways, an alternative approach to increase glycine availability will be needed as glycine administered exogenously may be utilized without increasing its availability for the biosynthesis of physiologically/metabolically important biomolecules. Currently, it is debatable whether obesity associated hypoglyciemia is a cause or consequence of insulin resistance (3, 4). Plasma glycine increases after bariatric surgery, returning to values within the normal range by 6-months post-surgery (17, 18). By studying the glycine metabolic pathways associated with the post-surgery increase in plasma glycine, we can better understand the relationship between abnormal glycine metabolism and insulin resistance and be more informed regarding the therapeutic potential of glycine-based treatment.

Our study had two main objectives: 1) to identify the glycine metabolic pathways that are dysregulated in obesity, and 2) to quantify the changes in these pathways when plasma glycine increases after bariatric surgery. For the first objective, we infused multiple isotopically labelled tracers to compare the rates of glycine flux, its oxidation, *de novo* synthesis, release from protein breakdown, and rate of disposal between participants with morbid obesity and participants with healthy weight (controls). As these kinetic parameters are the determinants of plasma glycine concentration, the differences between participants with morbid obesity and controls would allow us to identify the glycine metabolic pathways that are dysregulated by obesity. For the second objective, the participants with morbid obesity underwent bariatric surgery, and the glycine kinetic measurements were repeated 6-months post-surgery. This allowed us to confirm that these pathways were initially dysregulated in the obese state and to examine the temporal relationship between glycine metabolism and insulin resistance.

## Methods

### Study Participants

This study received approval from the SingHealth Centralized Institutional Review Board (CIRB Ref: 2018/2714), and all participants provided written informed consent. We recruited two groups of participants (n = 21 in each group). The first group consisted of individuals with morbid obesity who were scheduled for bariatric surgery, while the second group had individuals with healthy weights. Participants with morbid obesity were recruited from patients attending the Singapore General Hospital’s obesity clinic who were scheduled for bariatric surgery. They were recruited if they were between 21- and 65-years and had a BMI ≥ 32.5 kg/m^2^ with obesity-related complications. They were excluded if received insulin treatment, consumed excessive alcohol (> 1drink/day for females or > 2 drink/day for males), received systemic corticosteroid treatment, or had existing cardiovascular, kidney, or liver disorders. Individuals with healthy weight (BMI < 25 kg/m^2^) were recruited from our healthy volunteer database. These participants were age- and sex-matched and excluded if they had diabetes mellitus or any significant chronic medical illness. The full list of inclusion and exclusion criteria is listed in **Supplementary Table 1**.

At baseline, all participants underwent metabolic studies consisting of mixed-meal tolerance testing (MMTT), body composition analysis, and stable-isotope tracer infusion studies. As part of routine medical care, all participants with morbid obesity were seen by a dietician, and they were given dietary advice to reduce total energy intake by ~ 500 kcal/day while maintaining a balanced diet with 55% energy from carbohydrate, 15% from protein and 30% from fat. Participants with healthy weight did not receive any nutritional advice and were asked to maintain their habitual diet.

Participants with morbid obesity then underwent bariatric surgery and those who remained in the study were invited to returned at 6 months to evaluate the post-surgery changes in glycine kinetics and related metabolic parameters. Post-surgery dietary management was determined by the dietician and was in accordance with standard clinical guideline (19). Dietary energy requirements were individualized based on the subject’s post-surgery weight and estimated using the Harris-Benedict equation. Subjects were asked to consume adequate protein from healthy sources with a target of at least 1 g/kg/day, avoid consumption of simple carbohydrates, and increase the consumption of food rich in dietary fibers. In addition, subjects were prescribed elemental calcium 1000 mg/day, Vitamin D3 1000 IU/day, multivitamin tablets 2 capsules/day, and elemental iron 100 mg/day.

### Screening

All participants underwent a standard medical examination, anthropometric measurements, and blood sample collection after an 8-hour overnight fast. Screening blood test included Participants who fulfilled the recruitment criteria were then asked to return for the stable-isotopes infusion study on a separate day. Participants were also given a food diary to record their food intake prospectively for 5-days before their study visit, and the recordings were verified by a single clinical research coordinator. Total energy and macronutrient intakes were analyzed based on the local food database using the nutrient analysis software (Dietplan7, Frestfield Software, UK).

### Mixed-Meal Tolerance Testing

MMTT was performed to calculate indices of insulin sensitivity. During MMTT, a liquid meal (Ensure^©^Plus, Abbott Nutrition) was given at 6 kcal/kg (max 360 kcal). The liquid meal consisted of approximately 30% of energy from fat, 15% energy from protein, and 55% energy from carbohydrates. Blood samples were collected before and at 30, 60, 90, and 120 minutes after MMTT for plasma glucose and insulin measurements.

### Body Composition

Lean body mass (LBM), fat-free mass (FFM), and fat mass were measured using dual-energy X-ray absorptiometry (Hologic Discovery Wi densitometer, Hologic, Inc, Massachusetts, USA).

### Stable-Isotope Infusion Protocol

Stable isotope tracers: [1,2-^13^C_2_] glycine (99 atom% ^13^C), [2,3,3-^2^H_3_] serine (98 atom% ^2^H), [^2^H_5_] phenylalanine (98 atom% ^2^H), and NaH^13^CO_3_ (99 atom% ^13^C) were purchased as sterile and pyrogen free compounds (Cambridge Isotope Laboratories, MA), and reconstituted within 24-hours of the infusion.

Participants were asked to maintain their usual dietary habits and physical activity during the study period. They were asked to refrain from coffee, smoking, alcohol intake, and vigorous exercise (more than 1 hour of high-intensity physical activity) during the 24-hour before the study visit. To further limit variability in the duration of fasting and physical activity, all participants were admitted on the evening before the study visit and ate dinner prepared by the hospital’s kitchen (meal energy composition: 55% from carbohydrate, 33% from fat %, and 15% from protein). Participants subsequently fasted from 10.00 PM until the completion of the study protocol.

**Figure 1:**
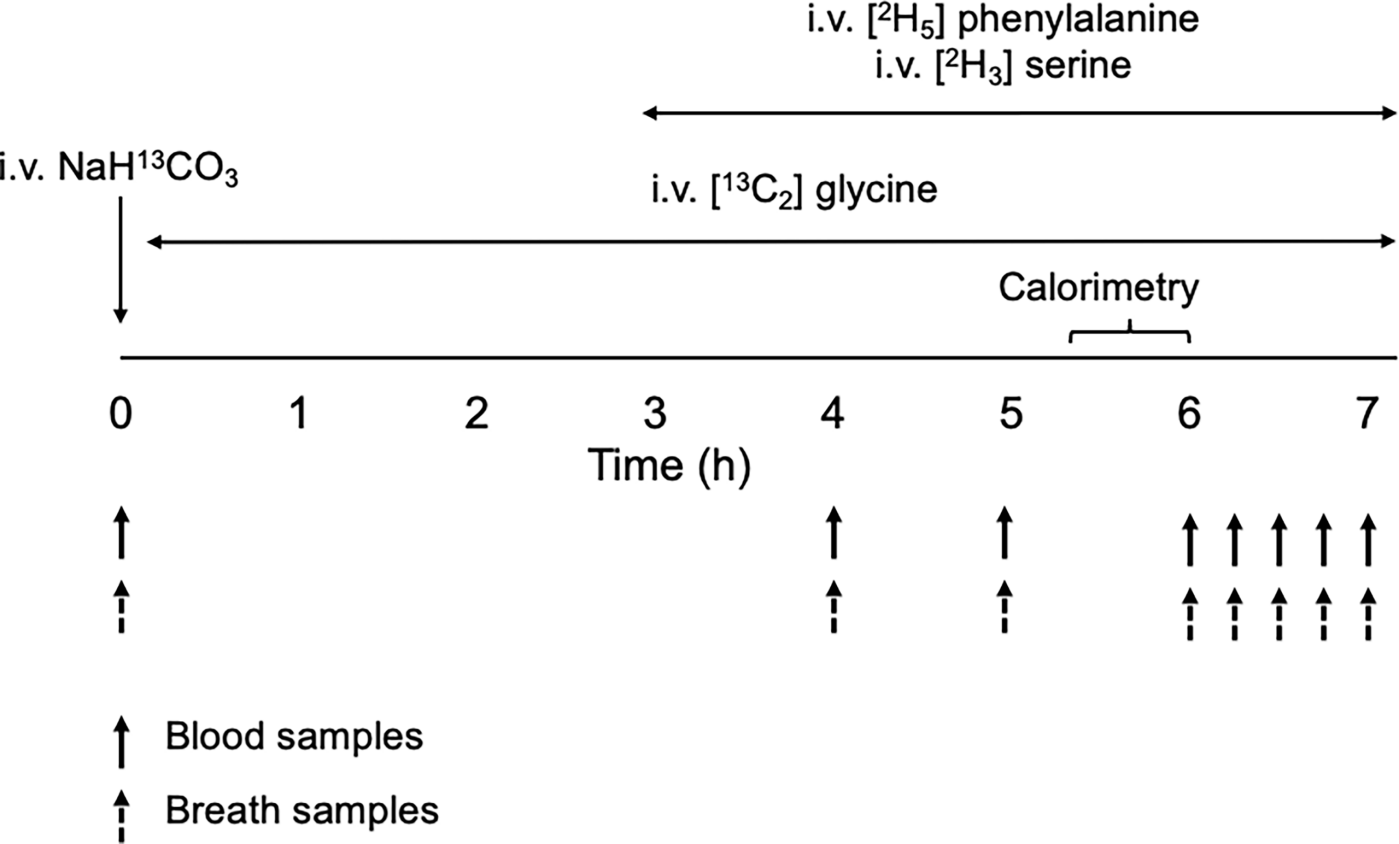
Schematic diagram of the stable-isotope infusion protocol for the measurement of glycine and serine kinetics.

Following an 8-h overnight fast, stable isotope tracers were infused as depicted in **Figure 1**. Two intravenous blood cannulas were first inserted on opposite arms: one for the infusion of tracers and the other for blood draws. A hand warmer was used to arterialize the venous blood collected. Fasting blood samples were collected for metabolite analyses and for background isotopic enrichments (IEs) of glycine, serine and phenylalanine. Breath samples were also collected for background IE of carbon dioxide. A bolus dose of NaH^13^CO_3_ (4 umol·kg FFM^-1^) was then injected to prime the bicarbonate pool with H^13^CO_3_ followed by a primed-constant infusion of [^13^C_2_] glycine (8 µmol·kgFFM^-1^, 8 µmol·kgFFM^-1^·h^-1^) for the next 7-hours. At the third hour of the infusion, intravenous primed-constant infusions of [^2^H_3_] serine (4 µmol·kgFFM^-1^, 4 µmol·kgFFM^-1^·h^-1^) and [^2^H_5_] phenylalanine (4 µmol·kgFFM^-1^, 4 µmol·kgFFM^-1^·h^-1^) were started and maintained for the next 4 hours. Additional blood and breath samples were taken simultaneously hourly from the 4^th^ to 6^th^ hours, then every 15 minutes during the last hour of the infusion (**Figure 1**). Carbon dioxide exhalation rate (VCO2) was measured using an indirect calorimeter (Quark RMR, Cosmed) between the 5.5 and 6th hour of the infusion.

### Sample Collection

Blood samples collected during screening were dispatched immediately for analysis at the clinical laboratory. Samples collected during the stable-isotope tracer studies were stored for batch analysis. The samples were first collected into pre-chilled EDTA tubes, immediately centrifuged at 4°C, and the plasma stored at -80°C. Breath samples were collected in a breath bag with a one-way valve, immediately transferred to a 10 mL evacuated glass tube, and stored at room temperature.

### Biochemical Analysis, Insulin, and Amino Acid Concentrations

Biochemical analyses were conducted at the Singapore General Hospital Clinical Biochemistry Laboratory, which is accredited by the College of American Pathologists. Standard biochemistry (creatine, liver panel, lipid profile, and insulin) were measured using immunoassay method (Abbott Architect i200; Abbott Diagnostics). HbA1c was measured using immunoassay (Roche Cobas c501 analyzer; Roche Diagnostics); this method is accredited by the National Glycoprotein Standardization Program and standardized to the Diabetes Control and Complications Trial assay. Plasma glucose concentration was measured using the glucose oxidase method (YSI Glucose Analyzer; YSI). Plasma amino acid concentrations were measured by ultra-performance liquid chromatography (ACQUITY H-Class System, Waters Corporation, MA, USA) using pre-column derivatization with 6-aminoquinolyl-N-hydroxysuccinimidyl carbamate (Waters AccQ×Tag™ assay kit, MA, USA) using norvaline (Sigma Aldrich, MO, USA) as internal standard. Plasma samples were deproteinized with 10% sulfosalicylic acid dihydrate, and derivatized using AccQ-Fluor™ derivative reagent. The derivatized AA were separated using gradient based ACQUITY UPLC BEH C18 column (130 Å, 1.7 µM, 2.1 mm x 150 mm) with ACQUIT UPLC Tunable UV (TUV) detector (6).

### Isotopic Enrichment (IE)

The IE of breath carbon dioxide was measured by isotope ratio mass spectrometry on a ThermoQuest Finnigan Delta + XL IRMS coupled with Gasbench-II. The plasma glycine, serine and phenylalanine isotopic enrichments were measured by liquid chromatography-tandem mass spectroscopy (LC-MS/MS). Briefly, plasma glycine, serine, and phenylalanine were converted into their DANS [5-(dimethylamino)-1-napthalene sulfonamide] derivatives and analyzed using a Kinetex C18 2.6µ 100 × 2.1 mm column (Phenomenex, Torrance, CA) on a triple quadrupole mass spectrometer (TSQ Vantage; Thermo Scientific, San Jose, CA), equipped with a HESI (heated-electrospray ionization) source, a Accela pump (Thermo Scientific) and a Thermal PAL autosampler (Thermo Scientific). The ions were then analyzed by SRM (selected reaction monitoring) mode. The transitions observed were precursor ions m/z 309, 310, and 311 to product ion m/z 170 for glycine, precursor ion m/z 339, 340, 341, 342 to product ion m/z 170 for serine and precursor ion m/z 399 and 404 to product ion m/z 120 and 125 for phenylalanine. Instrumental control, data acquisition, and analysis were performed by the XCalibur (version 2.1) software package (Thermo Scientific).

### Calculations

#### Insulin Resistance

Insulin resistance was calculated based on the Homeostatic Model Assessment for Insulin Resistance (HOMA-IR) (20),


$$\frac{fasting\;glucose\;\left(mmol/L\;\right)\;\;x\;fasting\;insulin\;\left(\text{μ}U/mL\right)}{22.5}$$

Insulin sensitivity was calculated using post-MMTT glucose and insulin values as the Matsuda index (21).


$$\frac{10,000}{\sqrt{\begin{array}{c} \left(fasing\;glucose\;\left[mg/dL]\;x\;fasting\;insulin\;[\mu U/mL\right]\;\right)(MMTT\;mean\;glucose\;\left[mg/dL\right]\\ \;x\;MMTT\;mean\;insulin\;\;\left[\mu U/mL\right]\;) \end{array}}}$$

The area under the curves (AUCs) for glucose and insulin following MMTT were also quantified using the trapezoidal rule.

#### Total Glycine or Serine Flux (Q)

Total glycine or serine flux (Q) represent turnover rates of glycine (Q_Gly_) or serine (Q_Ser_) and was calculated using the standard isotope dilution equation:


$$Total\;Q_{Gly\;or\;Ser}\;=\;\left(E_{i}/E_{p}\right)\;\;x\;I$$

Where *E_i_* is the isotopic enrichment of the infused [^13^C_2_] glycine or [^2^H_3]_ serine tracer, *E_p_* is the plateau isotopic enrichment of M+2 glycine or M+3 serine in plasma, and *I* is the infusion rate of the glycine or serine tracer. Endogenous glycine or serine flux was derived by subtracting the tracer infusion rate from total flux. The description of substrate flux in this article will refer to endogenous flux unless stated otherwise. Kinetic parameters were normalized to total body weight (μmol·kg^-1^·h^−1^) and LBM (μmol·kgLBM^−1^ ·h^−1^). Interpretation of kinetic parameters was the same regardless of how the data are expressed. Hence, kinetic data were presented in the main body of the paper as μmol·kg LBM^−1^ ·h^−1^ and in the supplemental files as per total body weight (μmol·kg^-1^·h^−1^).

#### Glycine Oxidation

Whole-body glycine oxidation was calculated based on the rate of ^13^CO_2_ excretion following constant infusion of [^13^C_2_] glycine tracer from the following equation:


$$\text{Glycine Ox}=\left(VCO_{2}\text{ x }E_{CO2}\text{ x }44.6\text{ x }60\right)/\left(\text{Weight x }0.78\text{ x }E_{Gly\;M+2}\text{ x }2\right)$$

Where *V*CO_2_ is the rate of CO_2_ exhalation (ml/min), *E*_CO2_ is the increase in isotopic enrichment over baseline of carbon dioxide in breath at steady state, and 0.78 corrects for bicarbonate that is not excreted during the experiment (22). *E*_Gly_ is the ^13^C (M+2) isotopic enrichment of plasma glycine. The terms 44.6 and 60 convert mL/min to mol/h; and the term 2 compensates for the formation of two ^13^CO_2_ molecules from the oxidation of one [^13^C_2_]-glycine molecule.

#### Glycine Non-Oxidative Disposal (NOD)

Glycine NOD represents the rate of glycine exiting the metabolic pool to enter proteins and biomolecules syntheses and conjugation pathways. It was calculated by subtracting the rate of glycine oxidation from total Q_Gly_:


$$\text{Glycine NOD}={\text{Total Q}}_{\text{Gly}}-\text{ Gly Ox}$$

#### *De Novo* Glycine or Serine Synthesis

During fasting, the flux of a dietary non-essential amino acid is comprised of its release from whole-body protein breakdown plus its *de novo* synthesis. Thus, *de novo* synthesis of glycine or serine was estimated by subtracting the contribution of whole-body protein breakdown from endogenous glycine or serine flux (End *Q_Gly or Ser_*).


$$De\;novo\text{ glycine}\left(\text{or serine}\right)\text{synthesis}={\text{ End Q}}_{\text{Gly or Ser}}-{\text{ PB}}_{\text{Gly or Ser}}$$

where PB_Gly or Ser_ represent the flux of glycine or serine from whole-body protein breakdown (calculated below). This was estimated by multiplying Q_Phe_ by 1.48 for glycine and 1.08 for serine, representing the estimated ratio of glycine or serine to phenylalanine released during whole-body protein breakdown. Our calculation takes into account amino acid flux from not only skeletal muscles but also from collagen and other organs, based on their relative contribution to whole-body protein turnover and the average content of glycine and serine per 100g of protein (23–25). The inclusion of amino acid flux from non-skeletal muscle organs is important as every 3^rd^ molecule of collagen is a glycine molecule, and collagen makes up 30% of whole-body protein.

#### Whole-Body Protein Breakdown

Phenylalanine is not synthesized endogenously; hence, phenylalanine flux during fasting was used to estimate whole-body protein breakdown as follows:


$$Q_{Phe}=I\text{ x }\left[\left(E_{i}/E_{p\;Phe}\right)-1\right]$$

Where *E_i_* and E*p* represent phenylalanine isotopic enrichment in the infusate and plasma, respectively. *I* is the tracer infusion rate, and the term -1 corrects for the contribution of the tracer infusion to the flux.

#### Serine Hydroxymethyltransferase (SHMT) Flux

Glycine and serine metabolism are closely linked through the interconversion of both amino acids by the enzyme SHMT. Both glycine synthesis from serine (*Q_Ser->Gly_)* and serine from glycine (*Q_Ser->Gly_*) contribute significantly to their *de novo* synthesis (12, 26, 27). *Q_Ser->Gly_* was estimated by monitoring the transfer of ^2^H label from the M+3 serine tracer to the metabolic product M+1 glycine:


$$Q_{Ser->Gly}=\;TotalQ_{Gly}\;x\;\left(E_{Gly\;M+1}/\;E_{Ser\;M+3}\right)$$

Where *E_Ser M+3_* is the IE of the infused [^2^H_3_] serine tracer in plasma and *E*_Gly M+1_ the IE of glycine derived from [^2^H_3_] serine in plasma.

Similarly, serine can be synthesized from glycine through SHMT either: 1) directly from glycine, producing M+2 serine from the [^13^C_2_] glycine tracer, or 2) indirectly to produce M+1 serine after the [^13^C_2_] glycine tracer is first converted to 5,10-methylenetetrahydrofolate (5,10-^13^CH2-THF) *via* the glycine cleavage system (GCS) reaction (27).


$$Q_{Gly->Ser\;M+2}=Q_{Ser}\;x\;(E_{Ser\;M+2}/\;E_{Gly\;M+2})$$

$$Q_{Gly->Ser\;M+1}=Q_{Ser}\;x\;\left(E_{Ser\;M+1}/\;E_{Gly\;M+2}\right)$$

Where *Q _Gly->Ser M+2_* represents serine synthesis from the direct conversion of M+2 glycine to M+2 serine and *E _Ser M+2_* is the isotopic enrichment of M+2 serine in plasma. *Q _Gly->Ser M+1_* represents serine synthesis from singly labeled glycine and *E_Ser M+1_* is the isotopic enrichment of M+1 serine in plasma. Total serine synthesis from glycine is the sum of Q _Gly->Ser M+2_ and Q _Gly->Ser M+1_

### Statistics

Our study’s primary outcome measurements were kinetic parameters of glycine metabolism. Therefore, we designed this study based on the assumption that any changes in plasma glycine concentration will be reflective of whole-body glycine kinetic parameters. Based on data from our earlier studies, we calculated that bariatric surgery increases plasma glycine concentration by 26 μmol/L (standard deviation = 26) (6, 17, 18). To obtain a power of 80% at a 0.05 significance level, a total sample size of 17 participants with morbid obesity undergoing bariatric surgery will need to be recruited. We recruited 21 subjects with morbid obesity (assuming a possible 20% drop out rate) to compare the changes in glycine kinetics before and after bariatric surgery. To compare the differences in baseline glycine kinetic parameters with controls, 21 participants with healthy weight were recruited.

As our data did not follow a normal distribution, non-parametric methods were selected for statistical testing and continuous data were presented as medians with interquartile range (IQR). Statistical differences between participants with morbid obesity and healthy weight were sought using the Mann–Whitney U test for continuous data and Fisher’s exact test for categorical data. Significant changes in glycine kinetic parameters after bariatric surgery were tested using Wilcoxon’s Signed Rank test. To examine the relationship between *de novo* glycine synthesis and plasma glycine concentration, we performed linear regression with log-transformed plasma glycine as the dependent variable and log-transformed *de novo* glycine synthesis as the independent variable. Since this study has two primary objectives, Bonferonni correction was used to account for the multiple comparisons and only two-tailed P values < 0.025 were considered statistically significant. Statistical testing was performed using STATA version 17 (StataCorp) and Prism version 9 (GraphPad Software Inc.).

## Results

### Baseline Characteristics of Study Participants

Details of subject recruitment are summarized in **Supplementary Figure 1**. Four participants with morbid obesity (3 females and 1 male) were lost to follow-up and were excluded from the post-surgery statistical analyses. As shown in **Table 1**, there was no difference in the median age of participants with morbid obesity and those with healthy weight. In participants with morbid obesity, 6 (28.6%) had type 2 diabetes, 10 (47.6%) hypertension, and 7 (35%) hyperlipidemia. All patients with diabetes were treated with metformin and half the patients with diabetes were taking 3 or more oral diabetes medications. In the patients with hypertension, calcium channel blockers were most used (60%), and 60% were taking two or more blood pressure lowering medications. The majority of patients with hyperlipidemia were taking statins (71%) and 57% were taking more than 1 lipid lowering medications (**Table 2**). None of the participants with healthy weight had any medical illness or consumed long-term medications. Participants with morbid obesity were placed on a reduced energy diet as part of routine clinical care (median 2.3 weeks) and there were no significant differences in their median total daily calorie intake at 1420 (882- 1660) kcal/day compared to the those with healthy weight at 1510 (1280- 1660) kcal/day, p = 0.5134. Similarly, dietary protein intake at 68.8 (52.3-76.4) vs. 60.7 (53.2- 76.6) g/day was not significantly different (p = 0.8116).

**Table 1:** Baseline characteristics of participants with healthy weight and with morbid obesity.

	Healthy weight (n = 21)	Morbid Obesity (n = 21)	*P* value
Age (years)	39.4 (31.3-47.2)	40.2 (32.2-46.3)	0.7462
Females, n (%)	16 (76.2%)	16 (76.2%)	1.00
Weight (kg)	55.4 (49.3- 61.1)	100 (93.4-114)	< 0.0001
BMI (kg/m2)	20.6 (19.4-22.5)	38.5 (35.3-43.3)	< 0.0001
Fat mass (kg)	17.9 (16.4-21.2)	48.6 (43.0-52.9)	< 0.0001
Lean body mass (kg)	35.1 (29.9- 38.1)	54.2 (44.4-64.6)	< 0.0001
Fat free mass (kg)	37.6 (32.1- 39.9)	55.9 (46.5- 67.2)	< 0.0001
Fat mass (%)	35.1 (28.4- 37.2)	45.7 (42.1- 50.7)	< 0.0001
Waist circumference (cm)	76 (74-82)	117 (112-122)	< 0.0001
Hip circumference (cm)	94 (90- 98)	126 (120-134)	< 0.0001
SBP (mmHg)	112 (102- 118)	121 (112-139)	0.0060
DBP (mmHg)	72 (65-75)	72 (69-81)	0.8371
Total cholesterol (mmol/L)	4.70 (4.12-5.49)	4.48 (3.85-5.61)	0.5010
HDL-C (mmol/L)	1.55 (1.35-1.72)	1.13 (0.99-1.24)	< 0.0001
Triglyceride (mmol/L)	0.60 (0.49- 0.90)	1.47 (1.23-1.81)	< 0.0001
LDL-C (mmol/L)	2.92 (2.34-3.45)	2.52 (2.19-3.71)	0.8276
Creatinine (µmol/L)	56 (50-67)	54 (47-62)	0.4427
Albumin (G/L)	39 (38-41)	38 (37-40)	0.4681
Bilirubin (umol/L)	13 (11-18)	11 (10-13)	0.0413
Alanine transaminase (U/L)	13 (10-19)	26 (17-38)	0.0013
Aspartate transaminase (U/L)	19 (18-22)	20 (18-64)	0.6934
Gamma-glutamyl transferase (U/L)	13 (12-21)	28 (24-37)	< 0.001

Values are median (inter-quartile range). The Mann–Whitney U test was used to test the statistical differences between participants with healthy weight and with morbid obesity. P value < 0.025 is considered as statistically significant. SBP, systolic blood pressure, DBP, diastolic blood pressure.

**Table 2:** Metabolic co-morbidities and medications in participants with healthy weight and with morbid obesity.

	Healthy weight	Morbid obesity	
	(n = 21)	Pre-surgery (n = 21)	Post-surgery (n = 17)
**Diabetes, n (%)**	0	6 (28.5)	1 (5.9)
Metformin, n	0	6	1
Sulphonylurea, n	0	3	0
DPP-IV inhibitor, n	0	2	0
GLP-1 agonist, n	0	0	0
SGL2 inhibitor, n	0	2	1
Alpha-glucosidase inhibitor, n	0	1	0
1 medication, n	0	3	0
2 medications, n	0	1	1
3 medications, n	0	1	0
≥ 4 medications, n	0	1	0
**Hypertension, n (%)**		10 (47.6)	6 (35.3)
Beta-blockers, n	0	3	2
ACE inhibitors/angiotensin II receptor blocker, n	0	6	3
Thiazide, n	0	2	1
Calcium channel blocker, n	0	7	4
1 medication, n	0	4	5
2 medications, n	0	5	0
≥ 3 medications, n	0	1	1
**Hyperlipidemia, n (%)**		7 (35)	2 (11.8)
Statins, n	0	5	2
Ezetimibe, n	0	1	0
Fibrates, n	0	1	0
1 medication, n	0	2	2
2 medications, n	0	4	0
3 medications, n	0	0	0

Compared to participants with healthy weight, participants with morbid obesity had significantly higher total body weight, BMI, fat mass, waist circumference, and hip circumference. Systolic blood pressure, serum alanine transaminase concentration, and triglyceride were higher, but HDL cholesterol lower in those with morbid obesity. Fasting blood glucose, HbA1C, and insulin concentrations were significantly higher in the participants with morbid obesity and they were significantly more insulin resistant with higher values of HOMA-IR and lower Matsuda index. In addition, AUC for insulin and glucose following MMTT were both significantly greater in the participants with morbid obesity (**Table 3**).

**Table 3:** Mixed-meal tolerance testing, insulin resistance indices in participants with healthy weight and with morbid obesity.

	Healthy weight (n = 21)	Morbid Obesity (n = 21)	*P* value
Fasting glucose (mg/dL)	87 (85- 89)	103 (90.7-127)	0.0017
HbA1C (%)	5.3 (5.1-5.5)	5.9 (5.6- 6.2)	< 0.0001
Fasting insulin (mU/L)	3.2 (2.8-4.7)	19.5 (13.6- 22.8)	< 0.0001
HOMA-IR	0.71 (0.55- 1.04)	4.70 (3.18-5.78)	< 0.0001
Matsuda Index	9.76 (7.71-12.30)	1.66 (1.34-2.52)	< 0.0001
Post-MMTT Insulin AUC	4530 (3840-5230)	12400 (9130-18200)	< 0.0001
Post-MMTT Glucose AUC	14500 (13300-17200)	19400 (16600-21400)	0.0001

Values are median (inter-quartile range). The Mann–Whitney U test was used to test the statistical differences between participants with healthy weight and with morbid obesity. P value < 0.025 is considered as statistically significant. MMTT, mixed-meal tolerance testing, AUC, area under curve.

### Baseline Plasma Amino Acid Concentrations and Substrate Kinetics

Compared to participants with healthy weight, those with morbid obesity had significantly lower plasma concentrations of glycine and serine (**Table 4** **).** By contrast, plasma concentrations of 6 of the 9 dietary essential amino acids (leucine, isoleucine, valine, phenylalanine, methionine, and lysine), and 5 non-essential amino acids (alanine, aspartate, glutamate, tyrosine, and cysteine) were higher in the participants with morbid obesity (**Table 4**).

**Table 4:** Plasma concentrations of amino acids in participants with healthy weight and with morbid obesity at baseline.

(µmol/L)	Healthy weight (n = 21)	Morbid Obesity (n = 21)	*P* value
**Non-essential**			
Glycine	201 (172-227)	167 (153-172)	0.0018
Serine	123 (112-135)	108 (89.4-120)	0.0079
Glutamine	443 (392-449)	453 (425-523)	0.1844
Cysteine	277 (231-300)	324 (306-362)	0.0001
Tyrosine	50.9 (44.6-54.9)	66.8 (61.5-69.4)	< 0.0001
Arginine	78.1 (67.5-89.4)	79.1 (72.8-97.1)	0.5333
Proline	129 (104-163)	148 (132-172)	0.1530
Alanine	226 (191-258)	315 (289-336)	< 0.0001
Asparagine	35.8 (30.3-37.9)	34.3 (30.2-38.0)	0.8813
Aspartate	1.93 (1.46-2.39)	2.61 (2.10-3.58)	0.0052
Glutamate	26.3 (17.2-34.4)	50.3 (43.3-73.3)	0.0003
**Essential**			
Leucine	109 (99-116)	139 (116-146)	0.0002
Isoleucine	50.0 (46.9-72.8)	67.2 (59.6-72.8)	< 0.0001
Valine	205 (182-229)	243 (221-288)	0.0016
Methionine	18.9 (17.4-21.2)	21.1 (20.1-24.3)	0.0028
Phenylalanine	57.1 (52.7-58.9)	64.6 (60.2-69.9)	< 0.0001
Threonine	105 (89-116)	116 (96.4-134)	0.1762
Lysine	161 (144-192)	195 (176-232)	0.0004
Histidine	76.6 (70.7-78.4)	68.6 (65.7-74.4)	0.0240
Tryptophan	38.2 (34.4-41.1)	39.0 (35.4-43.7)	0.7088

Values are median (inter-quartile range). The Mann–Whitney U test was used to test the statistical differences between participants with healthy weight and with morbid obesity. P value < 0.025 is considered as statistically significant.

**Figure 2:**
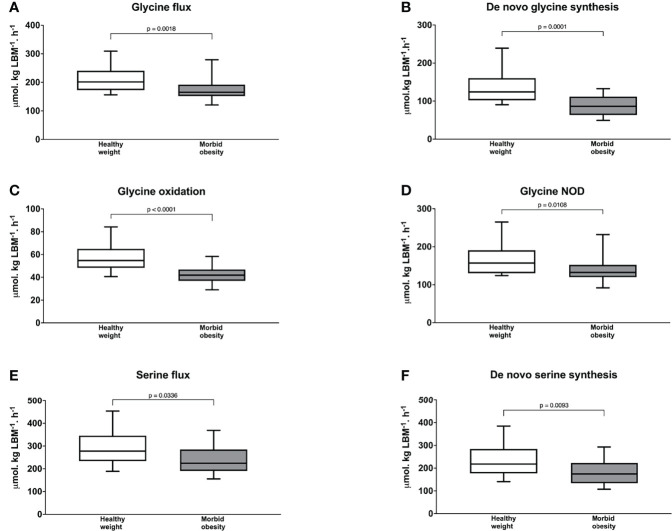
Glycine flux **(A)** *de novo* glycine synthesis **(B)** glycine oxidation **(C)** and glycine non-oxidative disposal **(D)** serine flux **(E)**; and *de novo* serine synthesis **(F)** in participants with healthy weight (n = 21) and with morbid obesity at baseline (n = 21). The Mann–Whitney U test was used to test the statistical differences between participants with healthy weight and participants with morbid obesity. NOD = non-oxidative disposal. *P* value < 0.025 is considered as statistically significant.

As shown in **Figure 2A**, participants with morbid obesity had significantly slower endogenous glycine flux at baseline at 165 (153-186) µmol·kg LBM^-1^·h^-1^ than those with healthy weight at 201 (173 -234), p = 0.0018 and this was associated with significantly slower *de novo* glycine synthesis at 86.2 (64.5-111) vs. 124 (103-159) µmol·kg LBM^-1^·h^1^, p = 0.0001(**Figure 2B**). In addition, glycine oxidation rate at 41.9 (37.5-46.0) vs. 54.7 (48.4-64.9) µmol·kgLBM^-1^·h^-1^, p < 0.0001 (**Figure 2C**) and NOD rate at 132 (121-152) vs. 157 (130 -184) µmol·kg LBM^-1^·h^-1^, p = 0.0108 (**Figure 2D**) were significantly slower in the participants with morbid obesity than those with healthy weight.

The direction of serine kinetics followed those of glycine. Endogenous serine flux was lower in participants with morbid obesity than those with healthy weight at 224 (197-283) vs. 278 (248-336) µmol·kg LBM^-1^·h^-1^ but this difference was not statistically different (p = 0.0336) (**Figure 2E**). Nonetheless, *de novo* serine synthesis (**Figure 2F**) was significantly slower with values of 174 (136-219) vs. 218 (177-284) µmol·kg LBM^-1^·h^-1^, p = 0.0093. Interpretation of serine and glycine kinetics were similar when these kinetic parameters were expressed as per total body weight. But, endogenous serine flux was significantly slower in participants with morbid obesity (**Supplementary Table 2**). No difference in phenylalanine flux was found between participants with morbid obesity and healthy weight, 53.5 (48.2-59.6) vs. 52.0 (47.0-57.6) µmol·kg LBM^-1^·h^-1^, p = 0.663.

### Post-Surgery Changes in Clinical Parameters

Among the 17 participants who remained in the study, 15 underwent sleeve gastrectomy and 2 Roux-en-Y gastric bypass. These participants returned for their post-surgery metabolic evaluation after a median of 6.4 (5.9-8.1) months, and their total energy intake at 784 kcal/day and protein intake at 50.2 g/day represent significant reductions compared to their baseline values (p < 0.001 for both). Post-surgery total body weight, BMI, fat mass, waist circumference, and hip circumference were all significantly lower than baseline values (p < 0.0001) (**Supplementary Table 3**).

Plasma triglyceride and alanine transaminase concentrations were lower post-surgery while HDL cholesterol was higher than baseline (p < 0.01 for all) (**Supplementary Table 2**). Fasting plasma glucose and HbA1C reduced significantly after bariatric surgery (p < 0.005 for both). These post-surgery changes were accompanied by significant improvements in insulin resistance, as demonstrated by the higher post-surgery Matsuda index, lower HOMA-IR, and a decrease in post-MTT glucose AUC (p < 0.01 for all) (**Supplementary Table 4**). The number of participants requiring medications and types of medications given for diabetes, blood pressure, and lipid treatment were decreased after surgery (**Table 2**).

### Post-Surgery Changes in Plasma Amino Acid Concentrations and Substrate Kinetics

**Figure 3:**
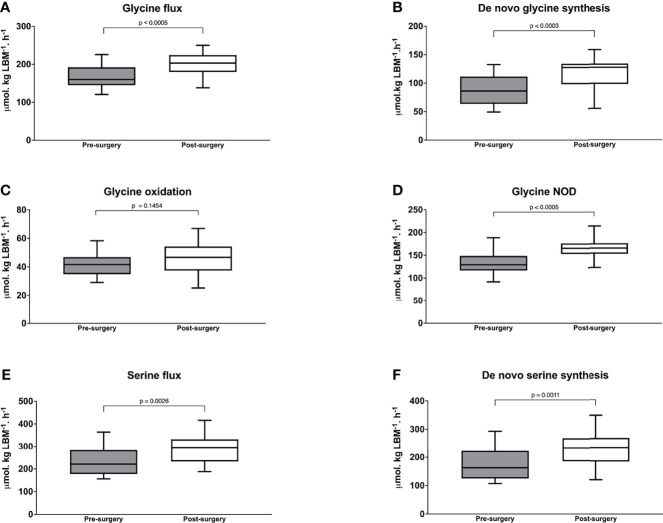
Glycine flux **(A)** *de novo* glycine synthesis **(B)** glycine oxidation **(C)** and glycine non-oxidative disposal **(D)** serine flux; and **(E)** *de novo* serine synthesis in with morbid obesity at baseline before (n = 17) and 6-months after bariatric surgery (n = 17). Wilcoxon’s Signed Rank test was used to determine the post-surgery changes. *P* value < 0.025 is considered as statistically significant.

**Figure 4:**
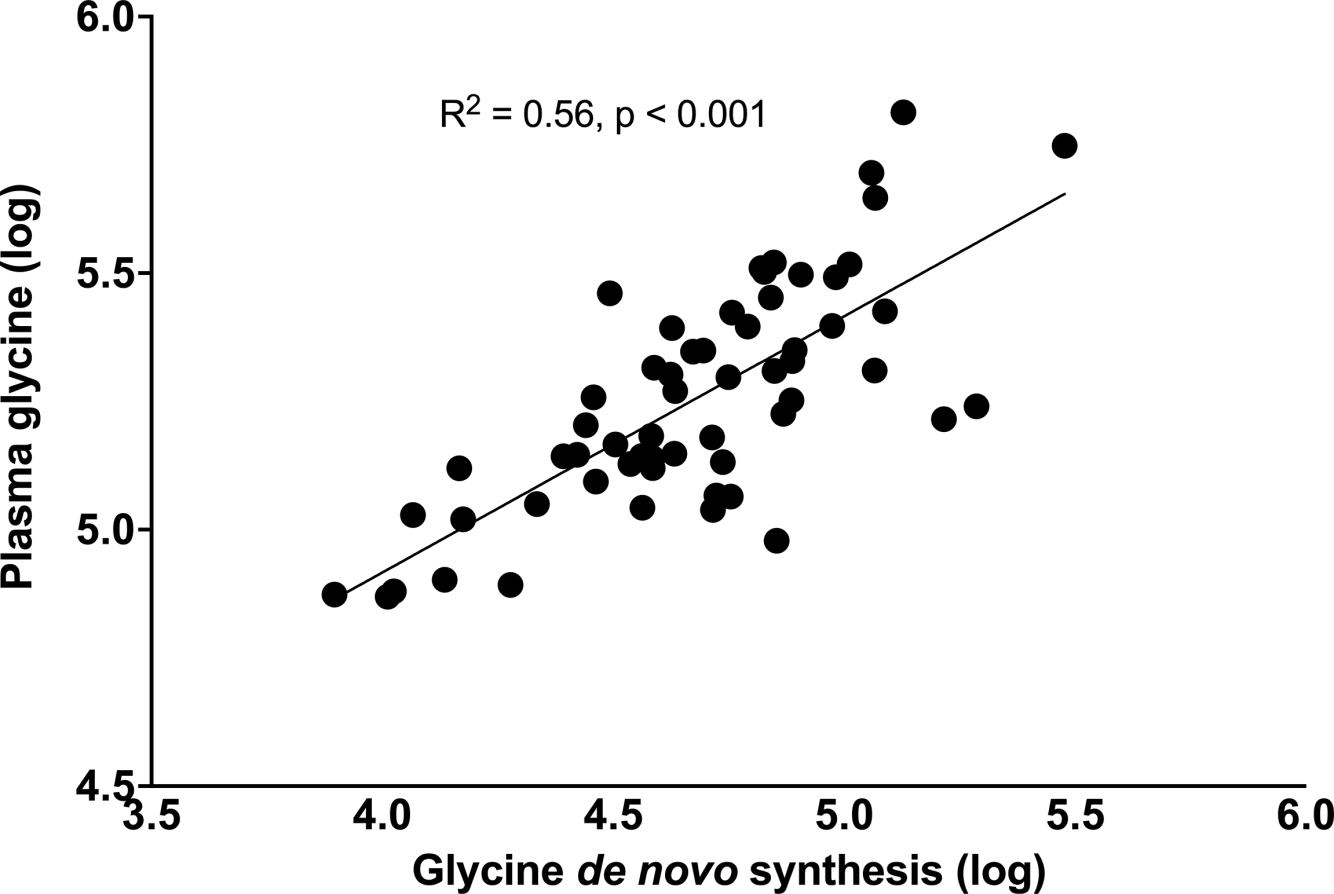
Relationship between plasma glycine concentrations with rates of glycine *de novo* synthesis. Data were log-transformed, and their association tested using the linear regression model.

Following surgery, plasma glycine and serine concentrations increased significantly to 210 (191-243) µmol/L, p < 0.0001 and 113 (101-134) µmol/L, p = 0.0056 respectively. By comparison, the blood concentration of 6 dietary essential amino acids and 5 non-essential amino acids deceased significantly (**Supplemental Table 5** **)**. Importantly, glycine flux increased significantly from 156 (150-186) to 203 (188-222) µmol·kg LBM^-1^·h^-1^, p < 0.001 and *de novo* glycine synthesis increased significantly from 86.2 (62.5-111) to 127 (98.3-133) µmol·kg LBM^-1^·h^-1^, p < 0.001 (**Figures 3A, B**). Glycine oxidation rate at 46.7 (37.9-53.9) µmol·kg LBM^-1^·h^-1^ was not significantly higher than the pre-surgery value of 41.6 (36.3-46.0) µmol·kg LBM^-1^·h^-1^, p = 0.1454 (**Figure 3C**). However, glycine NOD rate was higher at 165 (154-175) µmol·kg LBM^-1^·h^-1^ than the baseline value of 129 (121-145) µmol·kg LBM^-1^·h^-1^, p < 0.001 (**Figure 3D**). Post-surgery, serine flux also increased significantly from 221 (183-280) to 296 (239-311) µmol·kg LBM^-1^·h^-1^, p = 0.0026 (**Figure 3E**) and this was associated with an increase in *de novo* serine synthesis rate from 162.7 (125-223) to 234 (197-254) µmol·kg LBM^-1^·h^-1^, p < 0.0011 (**Figure 3F**). Similar results were obtained when glycine and serine kinetics were expressed as per kg total body weight, but the post-surgery glycine oxidation rate was significantly higher (**Supplementary Table 6**). Post-surgery phenylalanine flux at 57.2 (51.6 – 61.7) µmol·kg LBM^-1^·h^-1^ was not significantly different than baseline p = 0.3529. Regression analysis using the combined baseline and post-surgery parameters showed that the concentration of plasma glycine was significantly associated with the rate of glycine *de novo* synthesis (β = 0.50, p < 0.001, and model R^2^ = 0.56) (**Figure 4**).

### Substrate Flux *via* SHMT

At baseline, the rate of glycine flux derived from serine (*Q_Ser->Gly_*) per kg total body weight was significantly lower (p =0.0016) in the participants with morbid obesity compared to those with healthy weight (**Supplementary Table 7**), but statistical significance was lost when data was expressed per kg LBM (p = 0.1196). Rates of conversion of glycine to serine, either directly *via* SHMT (Q_Gly->Ser M+2_) or indirectly *via* 5,10-^13^CH2-THF (Q_Gly->Ser M+1_), were also significantly slower in participants with morbid obesity compared to those with healthy weight (p < 0.001 for both) (**Supplementary Table 7**).

Among the 17 participants who returned for their 6-month follow-up, post-surgery *Q_Ser->Gly_* increased significantly (p < 0.005). Q_Gly->Ser M+2_ and Q_Gly->Ser M+1_ were also significantly faster at following surgery (p <0.005) (**Supplementary Table 8**).

## Discussion

Abnormal plasma amino acid concentrations in obesity have been proposed as a predictor of poor cardiometabolic outcomes (28), propagator of insulin resistance (1), and potential therapeutic target (29). Recent scientific developments have focused on amino acids and metabolites that are elevated in the obese and insulin resistant states (30). By contrast, our current study focused on glycine - the simplest and smallest amino acid in the human body, but one that is consistently and paradoxically lower in individuals with obesity (3, 4). Plasma glycine may be low in obesity due to reductions in dietary intake, flux from protein breakdown, or *de novo* synthesis. Alternatively, obesity is a state of heightened metabolic demand, and there may be an increase in glycine catabolism or diversion of glycine to pathways of utilization. This paper presents the novel discovery that *de novo* glycine synthesis was impaired in participants with morbid obesity and that the increase in plasma glycine concentration after bariatric surgery was accompanied by a concordant increase in its endogenous synthesis. To the best of our knowledge, this is the first study that used stable-isotope tracers to measure glycine kinetics in individuals with morbid obesity directly and tracked the changes in metabolic flux through the glycine biosynthetic and disposal pathways after bariatric surgery.

Obesity is a risk factor for insulin resistance, and our study found that participants with morbid obesity had a higher HOMA-IR and lower Matsuda index than those with healthy weight at baseline. Furthermore, participants with morbid obesity had higher blood glucose values after consuming a mixed-meal, indicating significant impairment in insulin-mediated glucose uptake and utilization. Our study also showed significant differences in the baseline amino acid profiles between morbid obesity and healthy weight. The elevated plasma amino acids in the group with morbid obesity included the BCAAs, aromatic amino acids, methionine, alanine, and glutamate which are known metabolic signatures of insulin resistance (31). Similarly, the lower plasma glycine and serine concentrations in this group are recognized features associated with poor metabolic health (3, 4, 31). Following bariatric surgery, participants with morbid obesity lost weight and experienced significant improvements in insulin resistance and clinical indicators of glucose regulation. Importantly, plasma concentrations of amino acids elevated in the pre-surgery obese state decreased, while plasma glycine and serine concentrations increased significantly post-surgery. This pattern of changes in amino acids following bariatric surgery is consistent with reports in other lifestyle (32), surgical (33), and pharmacologic studies (34) that have targeted insulin resistance.

In the current study, participants with morbid obesity consumed the same amount of daily dietary protein as those with healthy weight at baseline, but they still had lower plasma glycine concentration. Hence, dietary protein intake is an unlikely explanation for hypoglycinemia. Further, the plasma concentrations of most dietary essential and many dietary non-essential amino acids were higher in the participants with morbid obesity. Importantly, despite a decrease in daily total dietary protein intake 6 months after bariatric surgery, plasma glycine concentrations increased significantly. Glycine flux from body protein breakdown was also unlikely a significant determinant of the final plasma glycine concentration as there were no differences in whole-body protein turnover rates between the groups at baseline or post-surgery.

As a nutritionally non-essential amino acid, it has been generally assumed that the daily metabolic demands for glycine can be met by its endogenous synthesis from various precursors. Hence, impairment of its *de novo* synthesis is usually not considered as an explanation for obesity-associated hypoglycinemia (3, 4). Indeed, studies have found that endogenous glycine synthesis can maintain glycine flux in children with severe protein energy malnutrition (35) and men consuming suboptimal amounts of dietary protein (36). However, because *de novo* glycine synthesis contributes as much as 80% of glycine flux in the fasting state (37), any compromise in its endogenous synthesis will have a quantitatively significant impact on its plasma concentration. In the current study, we found significantly slower *de novo* glycine synthesis in participants with morbid obesity compared to those with healthy weight. The reason why endogenous glycine synthesis was reduced in the participants with morbid obesity is unclear, but the increase in plasma glycine concentration and *de novo* glycine synthesis following bariatric surgery led us to speculate that this may be linked to insulin resistance and impaired glucose metabolism.

**Figure 5:**
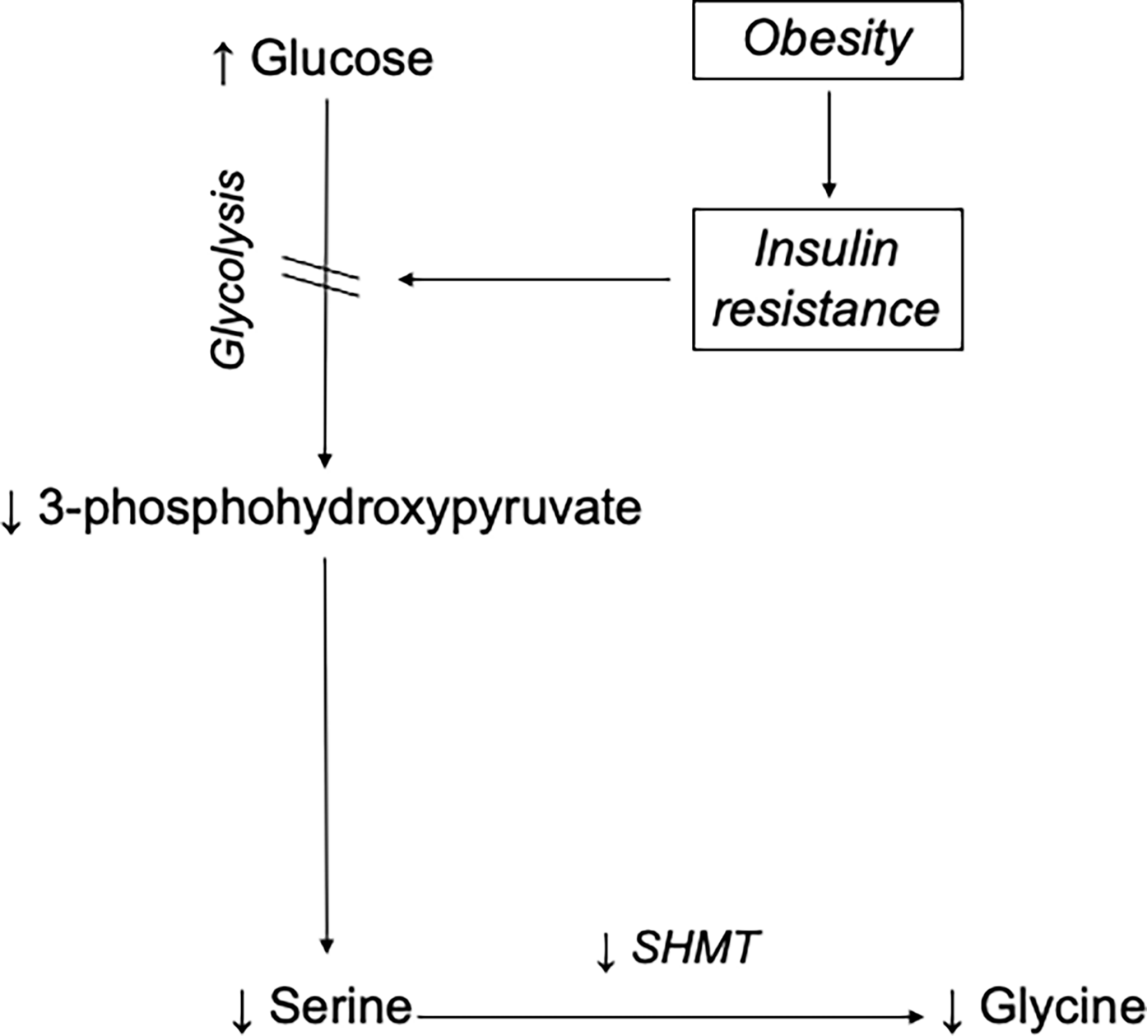
Mechanism for obesity-associated hypoglycinemia. This model proposes that obesity-induced insulin resistance impairs glucose uptake and results in high plasma glucose concentration. Consequently, glucose flux along the glycolytic pathway decreases, lowering the production of 3-phosphohydroxypyruvate, a major precursor for *de novo* serine synthesis. Since glycine is mainly synthesized from serine in human, a decreased supply of serine compromises SHMT-mediated *de novo* glycine synthesis. With time, the overall reduction in glycine production relative to its rate of utilization results in a lowering of its plasma concentration.

In humans, the major source is its synthesis from serine *via* the enzyme SHMT. Serine, in turn, can be derived from 3-Phosphoglycerate (3-PG), an intermediate of glucose metabolism (3, 4, 12). By tracing the transfer of stable-isotope labels between serine and glycine, we estimated the glycine synthesis from serine and confirmed that participants with morbid obesity had impaired glycine synthesis from serine *via* the SHMT reaction. The same was true for serine, whose *de novo* synthesis rate was significantly slower in participants with morbid obesity. We did not directly measure glycolysis, but impaired flux through the glycolytic pathway is a well-recognized feature of insulin resistance (38). This led us to speculate that 3-PG production was impaired in the participants with morbid obesity. Herein, we propose the hypothesis that obesity-induced insulin resistance disrupts cellular glucose uptake and hence, glycolytic flux thereby decreasing the production of 3-PG required for endogenous serine synthesis. In turn, the reduced serine synthesis compromises SHMT-mediated *de novo* glycine synthesis resulting in an overall reduction in glycine production relative to its rate of utilization and ultimately a lower plasma concentration (**Figure 5**).

Plasma glycine concentration in participants with morbid obesity can also be low due to an increase in glycine catabolism or the diversion of glycine to utilization pathways. An increase in glycine oxidation could cause hypoglycinemia, but our findings indicate that glycine oxidation rate was reduced in participants with morbid obesity than those with healthy weight. We believe that the lower glycine oxidation rate was consequent to the lower availability of glycine in the obese state. Increased conversion of glycine to serine can also lead to a decrease in circulating glycine. Our experimental data did not support this, as we found that the glycine-to-serine flux was lower in participants with morbid obesity compared to those with healthy weight.

Glycine is required in large quantities as a substrate to support the synthesis of body proteins. It constitutes 33% of structural proteins and several critical biomolecules necessary to maintain metabolic functions (e.g., purines, pyrimidines, GSH, porphyrin, and creatine). It is also a primary donor of 1-carbons and forms conjugates with potentially toxic metabolites such as endogenous (and xenobiotic) organic acids, derivatives of BCAAs, β-oxidation derived fatty acid intermediates, and metabolites of polyphenols (3, 4, 11, 12, 39). In obesity, hypoglycinemia can develop if large amounts of glycine are consumed for the biosynthesis of GSH or the conjugation and disposal of potentially toxic metabolites. Our method of measuring glycine NOD is not specific to any utilization pathway, but a significant increase in glycine consumption in these utilization pathways should result in higher glycine NOD rates which returns to normal post-bariatric surgery. Instead, we found that the opposite was true as whole-body glycine NOD rate was lower in participants with morbid obesity at baseline and increased post-surgery.

We did not design this study to provide a definitive answer to whether hypoglycinemia is a cause or consequence of insulin resistance in the obese state. Nonetheless, we found a potential metabolic link between impaired glucose metabolism with slower glycine synthesis. In addition, the trajectory of kinetic parameters in participants with morbid obesity after bariatric surgery plus results from recent Mendelian randomization studies suggest that hypoglycinemia is more likely a consequence of obesity-induced insulin resistance (40, 41). This finding may argue against developing a glycine-based treatment for clinical care. However, glycine remains a key molecule necessary for the maintenance of physiological and metabolic homeostasis in human health. Thus, regardless of the reason for why plasma glycine concentration is low in obesity, hypoglycinemia will adversely impact multiple bodily functions.

In obesity, lipolysis and lipid oxidation rates are increased, which leads to the generation of reactive oxidative species and oxidative stress (42). Various anti-oxidative defenses are activated in response, including the production of the principal intracellular anti-oxidative peptide glutathione to regulate systemic inflammation, redox homeostasis, and nitrosative stress. However, these defenses can be overwhelmed. Individuals with morbid obesity have lower plasma concentrations of glutathione and antioxidant enzyme activities, which is associated with a shift in redox status towards oxidation, and the accumulation of products of peroxidation damage and nitrosative stress (43). Such changes can disrupt normal physiology, including interfering with insulin signaling, glucose homeostasis, and contribute to the pathogenesis of obesity-related metabolic complications (42). Glycine, cysteine, and glutamate are precursors for glutathione synthesis, but only glycine was deficient in our study. Recent studies have identified low glycine availability as a rate-limiting factor for glutathione synthesis in humans and rodents with non-alcoholic fatty liver disease (NAFLD) (44, 45). Importantly, this was associated with the downregulation of genes involved with endogenous glycine synthesis and lipid oxidation, but the upregulation inflammation and fibrosis-related genes (44). Similarly, glycine supplementation of humans without morbid obesity but with low plasma glycine values improved glutathione availability and lowered systemic inflammation, oxidative, and nitrosative stress markers (46–49). In addition, glycine receptors are present in various inflammatory cells, and the activation of these receptors could result in additional anti-inflammatory, immunomodulatory and anti-apoptotic effects (50). Finally, glycine forms conjugates with potentially toxic endogenous and xenobiotic metabolites, and this pathway serves as an integral component of the human body’s detoxification system, which may be compromised when glycine availability is limited (1, 32).

Our study has several limitations. Based on the post-surgery reduction in insulin resistance and improvement in glycine metabolism, we argued that hypoglycinemia is a metabolic consequence of insulin resistance. However, some investigators have speculated that weight-independent factors also contribute to the post-bariatric surgery improvement in insulin resistance (51, 52). We could not be sure if the increase in glycine availability after bariatric surgery played a role as insulin resistance improved contemporaneously with other factors such as weight, body composition, and dietary carbohydrate intake. This uncertainty can be clarified by raising plasma glycine values directly and examining its effect on insulin resistance. Several human studies using dietary glycine supplementation have demonstrated promising results (46–48), but similar trials have not been conducted in individuals suffering from morbid obesity. As discussed earlier, pathways related to the utilization of glycine to synthesize glutathione and to form acylglycine conjugates may be affected by hypoglycinemia and exacerbate insulin resistance. However, we did not examine these pathways. Our study investigates glycine metabolism, including its kinetic interrelationships with serine metabolism at the whole-body level. It does not provide any information on the alteration in metabolism at a specific tissue/organ level. Dysregulated glycine metabolism at the tissue level would be important when considering cardiometabolic disorders such as NAFLD. Several subjects with morbid obesity did not return after bariatric surgery but were still included in the baseline comparison with controls. However, similar findings were obtained when the 4 participants who were lost to follow-up were excluded from the baseline statistical analyses.

In conclusion, we found that low plasma glycine concentration in participants with morbid obesity was associated with impaired *de novo* glycine synthesis. This finding implies that glycine can be regarded as a conditionally essential amino acid in obesity, and that plasma glycine concentration can be raised by simple measures such as dietary supplementation. However, the increase in plasma glycine concentration and its *de novo* synthesis after bariatric surgery plus similar changes in serine metabolism suggest that hypoglycinemia is a consequence of impaired glycolysis secondary to obesity-induced insulin resistance. As such, the metabolic benefit from correction of hypoglycinemia in individuals with obesity can be challenged and should be investigated in future intervention studies.

## Data Availability Statement

The raw data supporting the conclusions of this article will be made available by the authors, without undue reservation.

## Ethics Statement

The studies involving human participants were reviewed and approved by SingHealth Centralized Institutional Review Board. The patients/participants provided their written informed consent to participate in this study.

## Author Contributions

HT, FJ, JH, J-PK, and ET designed research; HT, JH, VW, and SC conducted research; HT, JH, and CL performed statistical analysis; HT, JH, and FJ wrote paper; HT and FJ had primary responsibility for final content. All authors read and approved the final manuscript.

## Funding

This study was supported by the National Medical Research Council Transition Award (grant reference: NMRC/TA/0063/2018).

## Conflict of Interest

The authors declare that the research was conducted in the absence of any commercial or financial relationships that could be construed as a potential conflict of interest.

## Publisher’s Note

All claims expressed in this article are solely those of the authors and do not necessarily represent those of their affiliated organizations, or those of the publisher, the editors and the reviewers. Any product that may be evaluated in this article, or claim that may be made by its manufacturer, is not guaranteed or endorsed by the publisher.

## Abbreviations

3-PG, 3-phosphoglycerate; AUC, Area under the curve; BCAAs, Branched-chain amino acids; GSH, glutathione; HOMA-IR, Homeostatic Model Assessment for Insulin Resistance; GCS, glycine cleavage system; Homeostatic Model Assessment for Insulin Resistance; IE, isotopic enrichment; LBM, Lean body mass; MMTT, mixed-meal tolerance testing; NOD, non-oxidative disposal; SHMT, Serine hydroxymethyltransferase.
