# Supplementary material for: De Novo Glycine Synthesis Is Reduced in Adults With Morbid Obesity and Increases Following Bariatric Surgery
Source: Front Endocrinol (Lausanne). 2022 Jun 9;13:900343. doi: 10.3389/fendo.2022.900343 (PMC9219591; doi:10.3389/fendo.2022.900343)
Supplement: Supplementary file 1 [file DataSheet_1.docx]

**Supplemental Figure 1**.

## Enrollment

Assessed for eligibility (n= 49)

Excluded (n= 7)

♦ Not meeting inclusion criteria (n= 3)

♦ Declined to participate (n = 1)

♦ Poor venous access (n= 3)

## Baseline

Morbid obesity (n= 21)

Healthy weight controls (n=21)

Bariatric surgery

Post-surgery evaluation (n=17)
♦Lost-to follow-up (n=4)

## 6-months

**Supplemental Table 1 Inclusion and exclusion criteria**

| **Inclusion criteria**  (Subject must meet all of the inclusion criteria to participate in this study) | **Exclusion criteria**  (All subjects meeting any of the exclusion criteria listed below at baseline will be excluded from participation) |
| --- | --- |
| 1. Age: 21-65 years | 1. Weight > 150 kg |
| 2. BMI < 25 kg/m^2^ for non-obese controls or BMI ≥ 32.5 kg/m^2^ for obese subjects scheduled for bariatric surgery | 2. Renal impairment (estimated creatinine clearance estimated by Cockcroft-Gault Equation < 60 ml/min) |
| 3. Able to provide informed consent | 3. Haemoglobin concentration < 10 g/L |
|  | 4. Serum alanine aminotransferase or aspartate aminotransferase above 2x upper limit of normal |
|  | 5. Uncontrolled hypertension (BP > 180/110 mmHg) |
|  | 6. Pregnancy |
|  | 7. Nursing mothers |
|  | . Significant cardiovascular disease  (e.g. acute myocardial infarction, congestive cardiac failure, ischemic heart diseaes, atrial fibrillation, sick sinus syndrome, supraventricular tachycardia) |
|  | 8. Previous stroke |
|  | 9. Uncontrolled thyroid disease |
|  | 10. Surgery requiring general anaesthesia within 4-weeks before enrolment |
|  | 11. Psychiatric disorders requiring medication |
|  | 12. Significant alcohol intake (> 1 unit per day for women and > 2 units per day for men) |
|  | 13. Subcutaneous insulin injections |
|  | 14. Systemic steroid usage (eg. prednisoline, hydrocortisone, cortisone, dexamethasone) |
|  | 15. Cancer within the last 5-years (except squamous cell and basal cell cancer of the skin) |
|  | 16. Any factors likely to limit adherence to study protocol (e.g., dementia; alcohol or substance abuse; history of unreliability in medication taking or appointment keeping; significant concerns about participation in the study from spouse, significant other or family members |
|  | 16. Known diabetes mellitus (only for controls) |
|  |  |

**Supplemental Table 2 Glycine and serine kinetics of participants with healthy weight and with morbid obesity at baseline expressed per kg total body weight**

|  | **Healthy-weight**  **(n = 21)** | **Morbid Obesity**  **(n = 21)** | ***P value*** |
| --- | --- | --- | --- |
| **Per kg body weight (µmol . kg ^-1^ . h^-1^)** | | | |
| Glycine endogenous flux | 129  (117-140) | 86.7  (74.9-95.8) | <0.0001 |
| Glycine Oxidation | 34.2  (31.6-38.7) | 21.9  (20.2-24.1) | <0.0001 |
| Glycine Non-oxidative disposal | 99.7  (87.7-106) | 71.1  (59.4-74.1) | <0.0001 |
| Glycine de novo synthesis | 78.7  (69.5-91.6) | 43.2  (36.1-56.6) | <0.0001 |
| Serine Flux | 178  (156-198) | 126  (103-141) | <0.0001 |
| Serine de novo synthesis | 141  (122-168) | 92.0  (70.2-114) | <0.0001 |

Values are median (inter-quartile range). The Mann–Whitney U test was used to test the statistical differences between participants with healthy weight and participants with morbid obesity. *P* value < 0.025 is considered as statistically significant.

**Supplemental Table 3 Clinical parameters of participants with morbid obesity at baseline and 6-months after bariatric surgery**

|  | **Pre-surgery**  **(n = 17)** | **Post-surgery**  **(n = 17)** | ***P* value** |
| --- | --- | --- | --- |
| Weight (kg) | 100.8  (93.5-122.6) | 75.4  (73.0-91.3) | < 0.0001 |
| BMI (kg/m^2^) | 40.0  (35.6-43.3) | 29.9  (27.4-32.8) | < 0.0001 |
| Fat mass (kg) | 49.9  (43.3-52.9) | 32.7  (28.9-35.6) | < 0.0001 |
| Lean body mass (kg) | 54.2  (44.4-65.4) | 42.4  (38.5-50.9) | 0.0002 |
| Fat free mass (kg) | 55.9  (46.5- 68.0) | 44.5  (41.1- 53.9) | 0.0002 |
| Fat mass (%) | 45.7  (42.3- 49.2) | 43.1  (34.3-44.1) | 0.0018 |
| Waist circumference (cm) | 120  (112-131) | 98  (94-111) | < 0.0001 |
| Hip circumference (cm) | 127  (123-134) | 108  (105-115) | < 0.0001 |
| SBP (mmHg) | 121  (112-139) | 116  (103-120) | 0.1006 |
| DBP (mmHg) | 72  (69-81) | 67  (64- 75) | 0.1486 |
| Total cholesterol (mmol/L) | 4.48  (3.83-5.61) | 4.79  (3.8- 5.18) | 0.5477 |
| HDL-C (mmol/L) | 1.06  (0.95-1.23) | 1.23  (1.01-1.38) | 0.0031 |
| Triglyceride (mmol/L) | 1.40  (1.23-1.73) | 0.95  (0.77- 1.20) | 0.0026 |
| LDL-C (mmol/L) | 2.55  (2.19-3.81) | 3.11  (2.38- 3.35) | 0.7467 |
| Creatinine (µmol/L) | 54  (47-62) | 58  (46-61) | 0.3340 |
| Albumin (G/L) | 38  (37-40) | 37  (36-38) | 0.0022 |
| Bilirubin (umol/L) | 11  (10-13) | 12  (11-17) | 0.0276 |
| Alkaline Phosphatase (U/L) | 12  (9-19) | 17.5*  (9.5-23) | 0.1629 |
| Alanine transaminase (U/L) | 26  (17-32) | 14  (11-17) | 0.0055 |
| Aspartate transaminase (U/L) | 20  (18-24) | 18  (16-21) | 0.4234 |

Values are median (inter-quartile range). Wilcoxon’s Signed Rank test was used to determine the post-surgery changes in participants with morbid obesity. SBP = systolic blood pressure, DBP = diastolic blood pressure, * ALP for 1 subject after surgery was below the range of detection. ** folate for 11 post-op and 6 pre-op were above the detection range. *P* value < 0.025 is considered as statistically significant.

**Supplemental Table 4 Insulin resistance and glucose homeostasis parameters based on fasting and post-mixed meal tolerance test in participants with morbid obesity at baseline and 6-months after bariatric surgery**

|  | **Pre-surgery**  **(n = 17)** | **Post-surgery**  **(n = 17)** | ***P* value** |
| --- | --- | --- | --- |
| Fasting glucose (mg/dL) | 102  (92-116) | 94  (79-98) | 0.0032 |
| HbA1C (%) | 5.8  (5.3- 6.1) | 5.1  (4.9-5.4) | 0.0025 |
| Fasting insulin (mU/L) | 19.5  (13.1- 21.0) | 5.1  (3.2-6.1) | < 0.0001 |
| HOMA-IR | 4.70  (3.07-5.74) | 1.20  (0.68-1.5) | < 0.0001 |
| Matsuda Index | 1.68  (1.5-2.52) | 4.85  (3.50-5.75) | 0.0001 |
| Post-MMTT Insulin AUC | 12100  (7660-17800) | 11300  (8560-12500) | 0.1454 |
| Post-MMTT Glucose AUC | 19400  (16600-21400) | 16000  (14200-18900) | 0.0017 |

Values are median (inter-quartile range). Wilcoxon’s Signed Rank test was used to determine the post-surgery changes. MMTT = mixed-meal tolerance testing, AUC = area under curve. *P* value < 0.025 is considered as statistically significant.

**Supplemental Table 5 Plasma concentrations of amino acids in participants with morbid obesity before and 6-months after bariatric surgery**

| **(µmol/L)** | **Pre-surgery**  **(n = 17)** | **Post-surgery**  **(n = 17)** | ***P* value** |
| --- | --- | --- | --- |
| **Non-essential** |  |  |  |
| Glycine | 163  (153-171) | 210  (191-243) | < 0.0001 |
| Serine | 105  (88-120) | 113  (101-134) | 0.0056 |
| Glutamine | 453  (425-523) | 473  (443-496) | 0.8900 |
| Cysteine | 315  (306-352) | 339  (306-357) | 0.9632 |
| Tyrosine | 66  (62-71) | 50  (49-52) | 0.0001 |
| Arginine | 79  (74-95) | 82  (76-94) | 0.7119 |
| Proline | 144  (126-153) | 128  (119-139) | 0.0056 |
| Alanine | 314  (289-329) | 245  (222-268) | 0.0001 |
| Asparagine | 33  (30-38) | 32  (31-38) | 0.5477 |
| Aspartate | 3  (2-4) | 2  (1-2) | 0.0026 |
| Glutamate | 50  (35-73) | 25  (19-36) | 0.0004 |
| **Essential** |  |  |  |
| Leucine | 130  (115-143) | 118  (109-129) | 0.0348 |
| Isoleucine | 65  (60-71) | 55  (52-58) | 0.0003 |
| Valine | 237  (221-281) | 221  (207-237) | 0.0129 |
| Methionine | 21  (20-23) | 19  (18-20) | < 0.0001 |
| Phenylalanine | 65  (61-69) | 55  (53-60) | < 0.0001 |
| Threonine | 103  (95-132) | 102  (81-124) | 0.4586 |
| Lysine | 190  (173-219) | 185  (169-190) | 0.0448 |
| Histidine | 69  (66-74) | 73  (70-79) | 0.0984 |
| Tryptophan | 39  (37-44) | 37  (34-38) | 0.0079 |

Values are median (inter-quartile range). Wilcoxon’s Signed Rank test was used to determine the post-surgery changes**.** *P* value < 0.05 is considered as statistically significant.

**Supplemental Table 6 Glycine and serine kinetics of participants with morbid obesity at baseline and 6-months after bariatric surgery normalized total body weight**

| **Per kg body weight (µmol . kg ^-1^ . h^-1^)** | **Pre-surgery**  **(n = 17)** | **Post-surgery**  **(n = 17)** | ***P value*** |
| --- | --- | --- | --- |
| Glycine endogenous flux | 84.5  (73.7-94.2) | 119  (99.4-128) | <0.0001 |
| Glycine Oxidation | 21.0  (19.2-24.1) | 26.7  (21.9-33.8) | 0.0110 |
| Glycine Non-oxidative disposal | 70.7  (58.8-73.3) | 93.4  (77.8-106) | <0.0001 |
| Glycine de novo synthesis | 43.0  (33.0-56.7) | 70.5  (55.-83.4) | 0.0002 |
| Serine endogenous flux | 117  (96.6-138) | 163  (140-189) | 0.0001 |
| Serine de novo synthesis | 85.7  (70.0-111) | 130  (108-151) | 0.0002 |

Values are median (inter-quartile range). Wilcoxon’s Signed Rank test was used to determine post-surgery changes. *P* value < 0.025 is considered as statistically significant.

**Supplemental Table 7 Substrate flux via the serine hydroxymethyltransferase reaction in participants with healthy weight and with morbid obesity expressed as per kg lean body mass and total body weight**

|  | **Healthy weight**  **(n = 21)** | **Morbid obesity**  **(n = 21)** | ***P value*** |
| --- | --- | --- | --- |
| **Per kg LBM per hour (µmol . kg LBM^-1^ . h^-1^)** | | | |
| Q_Ser->Gly (M+1)_ | 53.1 (39.6-72.3) | 44.4 (35.4-60.6) | 0.1196 |
| Q_Gly>Ser (M+1)_ | 49.1  (43.4-57.6) | 38.5  (24.4-43.6) | 0.0031 |
| Q_Gly>Ser (M+2)_ | 127  (92.9-159) | 83.1  (67.5-110) | 0.0022 |
| **Per kg body weight (µmol . kg^-1^ . h^-1^)** | | | |
| Q_Ser->Gly (M+1)_ | 32.5  (27.3-43.9) | 22.3  (18.6-28.8) | 0.0016 |
| Q_Gly>Ser (M+1)_ | 31.7  (26.0-35.4) | 18.1  (14.0-21.9) | <0.0001 |
| Q_Gly>Ser (M+2)_ | 78.7  (60.5-89.0) | 43.8  (26.1-60.5) | <0.0001 |

Values are median (inter-quartile range). *The Mann–Whitney U test was used to test the statistical differences between participants with healthy weight and participants with morbid obesity. *P* value < 0.025 is considered as statistically significant. **

**Supplemental Table 8 SHMT flux of participants with morbid obesity at baseline and 6-months after bariatric surgery expressed as per kg lean body mass and total body weight**

|  | **Pre-surgery**  **(n = 17)** | **Post-surgery**  **(n = 17)** | ***P value*** |
| --- | --- | --- | --- |
| **Per kg LBM per hour (µmol . kg LBM^-1^ . h^-1^)** | | | |
| Q_Ser->Gly (M+1)_ | 42.4 (35.4-50.4) | 56.6 (45.3-65.0) | 0.0026 |
| Q_Gly>Ser (M+1)_ | 38.5  (24.3-43.6) | 47.0  (36.7-58.8) | 0.0013 |
| Q_Gly>Ser (M+2)_ | 82.3  (59.5-110) | 120  (101-139.2) | 0.0011 |
| **Per kg body weight (µmol . kg^-1^ . h^-1^)** | | | |
| Q_Ser->Gly (M+1)_ | 22.3  (18.1-28.3) | 33.0  (28.1-41.2) | 0.0007 |
| Q_Gly>Ser (M+1)_ | 17.5  (13.6-21.9) | 27.3  (20.3-35.1) | 0.0002 |
| Q_Gly>Ser (M+2)_ | 41.5  (32.2-56.1) | 67.2  (55.4-77.2) | <0.0001 |

Values are median (inter-quartile range). Wilcoxon’s Signed Rank test was used to determine post-surgery changes. *P* value < 0.05 is considered as statistically significant.
